# Mortality burden from seasonal influenza in Chongqing, China, 2012–2018

**DOI:** 10.1080/21645515.2019.1693721

**Published:** 2020-04-28

**Authors:** Li Qi, Qin Li, Xian-Bin Ding, Yuan Gao, Hua Ling, Tian Liu, Yu Xiong, Kun Su, Wen-Ge Tang, Lu-Zhao Feng, Qi-Yong Liu

**Affiliations:** a State Key Laboratory of Infectious Disease Prevention and Control, Collaborative Innovation Center for Diagnosis and Treatment of Infectious Diseases, National Institute for Communicable Disease Control and Prevention, Chinese Center for Disease Control and Prevention, Beijing, China; b Infectious Disease Control and Prevention Department, Chongqing Municipal Center for Disease Control and Prevention, Chongqing, China; c Infectious Disease Control and Prevention Department, Jingzhou Center for Disease Control and Prevention, Jingzhou City, Hubei Province, China; d Key Laboratory of Surveillance and Early-warning on Infectious Disease, Division of Infectious Disease, Chinese Center for Disease Control and Prevention, Beijing, China

**Keywords:** Influenza, excess mortality, burden

## Abstract

**Purpose:**

To estimate influenza-associated excess mortality rates (EMRs) in Chongqing from 2012 to 2018.

**Methods:**

We obtained weekly mortality data for all-cause and four underlying causes of death (circulatory and respiratory disease (CRD), pneumonia and influenza (P&I), chronic obstructive pulmonary disease (COPD) and ischemic heart disease (IDH)), and influenza surveillance data, from 2012 to 2018. A negative-binomial regression model was used to estimate influenza-associated EMRs in two age groups (<65 years and ≥65 years).

**Results:**

It was estimated that an annual average of 10025 influenza-associated deaths occurred in Chongqing, corresponding to 5.2% of all deaths. The average EMR for all-cause death associated with influenza was 33.5 (95% confidence interval (*CI*): 31.5–35.6) per 100 000 persons, and in separate cause-specific models we attributed 24.7 (95% *CI*: 23.3–26.0), 0.8 (95% *CI*: 0.7–0.8), 8.5 (95% *CI*: 8.1–9.0) and 5.0 (95% *CI*: 4.7–5.3) per 100 000 persons EMRs to CRD, P&I, COPD and IDH, respectively. The estimated EMR for influenza B virus was 20.6 (95% *CI*: 20.3–21.0), which was significantly higher than the rates of 5.3 (95% *CI*: 4.5–6.1) and 7.5 (95% *CI*: 6.7–8.3) for A(H3N2) and A(H1N1) pdm09 virus, respectively. The estimated EMR was 152.3 (95% *CI*: 136.1–168.4) for people aged ≥65 years, which was significantly higher than the rate for those aged <65 years (6.8, 95% *CI*: 6.3–7.2).

**Conclusions:**

Influenza was associated with substantial EMRs in Chongqing, especially among elderly people. Influenza B virus caused a relatively higher excess mortality impact compared with A(H1N1)pdm09 and A(H3N2). It is advisable to optimize future seasonal influenza vaccine reimbursement policy in Chongqing to curb disease burden.

## Introduction

Globally, seasonal influenza has been associated with substantial morbidity and mortality every year. At the global level, influenza has been estimated to cause approximately 290 000–650 000 respiratory deaths annually,^1^ which was higher than previous estimates-roughly 250 000–500 000 respiratory and circulatory deaths each year.^2^

Estimation of influenza-associated mortality burden is important for understanding the epidemiology of influenza, guiding vaccination programs, evaluating the use of diagnostic tests and antiviral drugs, and planning for seasonal epidemics and future pandemics. However, estimating the mortality burden associated with influenza remains challenging due to the following challenges: first, influenza diagnosis is usually based on symptoms and laboratory confirmation is not routinely conducted in hospitals; second, many deaths that may be caused by influenza occur after virus can be detected; third, influenza is rarely recorded as the cause of death in the death registration system. Therefore, directly counting influenza deaths usually grossly underestimate the mortality burden of influenza. To overcome underestimation of influenza-related deaths, various statistical models have been employed to estimate the mortality burden associated with influenza,^1,3,4^ among which negative-binomial regression model has been widely used.^5,6^ The rationale for the modeling approach was to estimate influenza-associated excess mortality rate (EMR), which was defined as the difference between observed and expected mortality based on the regression model in the absence of influenza.

**Figure 1.**
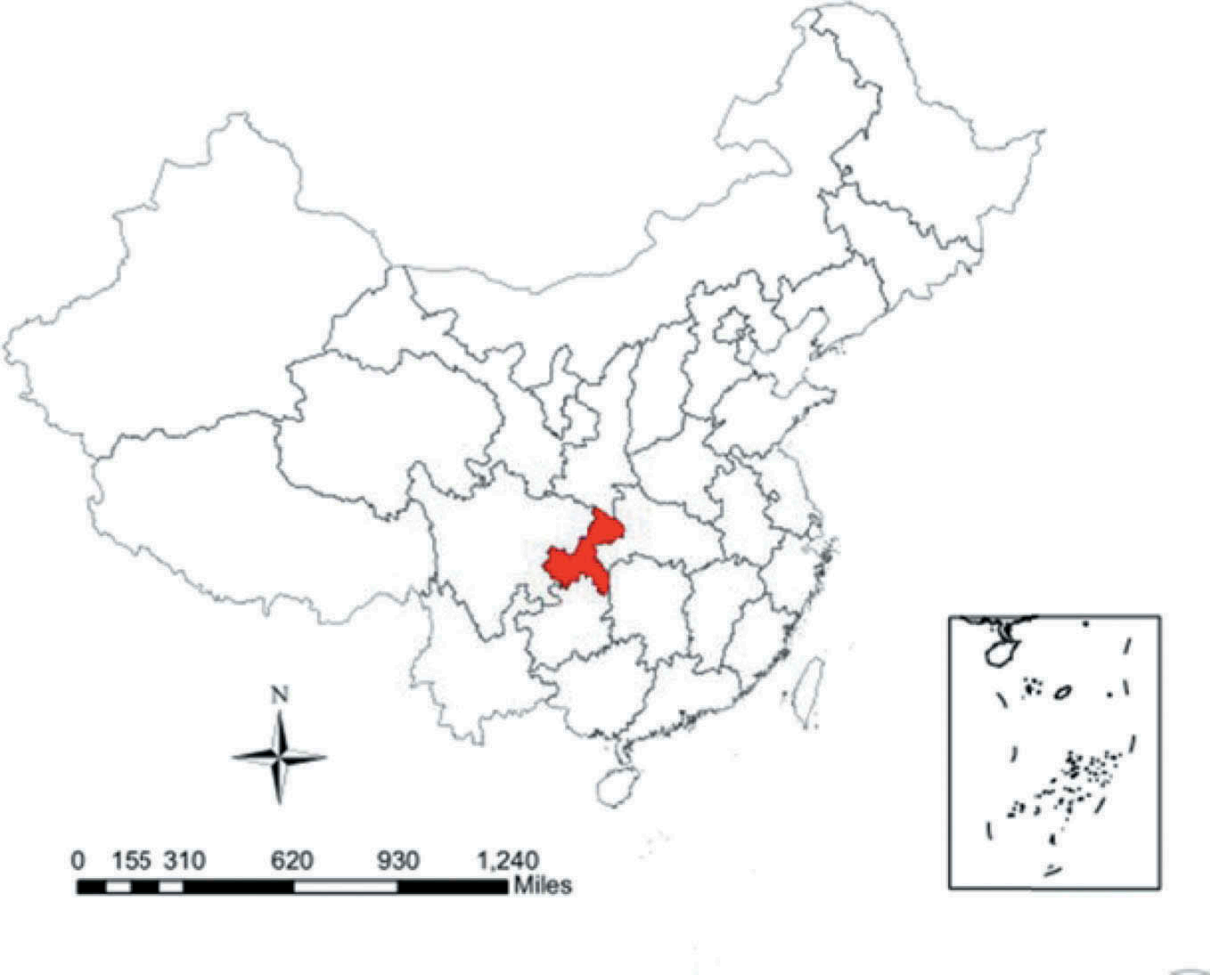
The geographical location of Chongqing, China.

Considering the diverse seasonality patterns, income levels, and healthcare access, influenza mortality burden varies across geographical locations.^1,4,7^ With the latitude of 29.6°N and a subtropical climate with four distinct seasons, Chongqing is the largest municipality with over 30 million registered inhabitants, which is located in Southwestern China (Figure 1). Previous study demonstrated that influenza virus circulated throughout the year and showed two possible peaks.^8^ However, little is known about the impact of influenza on death in this area.

To address this gap, we applied a negative-binomial model to the weekly counts of deaths and influenza viral data to estimate the mortality burden of influenza in Chongqing by death category, age group, and influenza type/subtypes during the period of 2012–2018.

## Materials and methods

### Mortality data and population denominators

According to China Center for Disease Control and Prevention (CDC), every individual death certificate in China is required to be registered in the death registration system. The causes of mortality are coded according to the International Classification of Diseases, the 10^th^ version (ICD-10). Coding practices are based on a standardized protocol, and quality control is conducted routinely by staff from the local CDC. We obtained separate data for five underlying causes of death in Chongqing from 2012 to 2018: circulatory and respiratory disease (CRD, ICD-10: J00-J99 or I00-I99), pneumonia and influenza (P&I, ICD-10: J10-J18), chronic obstructive pulmonary disease (COPD, ICD-10: J40-J47), ischemic heart disease (IHD, ICD-10: I20-I25), as well as total deaths.

The annual population data were obtained from the Household Registration Department of Chongqing Municipal Statistic Bureau. EMR was stratified by death category, influenza type/subtypes and two age-groups (0–64, and ≥65 years).

### Influenza virological surveillance

Influenza surveillance was conducted in seven sentinel hospitals throughout the year in Chongqing from 2012 to 2018. The seven sentinel hospitals were selected based on high accessibility among patients, high qualification of medical staff, adequate specimen storage capacity, and the desire of the physicians and nurses to participate voluntarily in the surveillance program. Surveillance is conducted in sentinel hospital emergency rooms and internal medicine and pediatric outpatient departments. The influenza surveillance protocol in Chongqing is in accord with the national influenza surveillance protocol and has been stated in a previous study.^8^ We obtained influenza virological surveillance data from seven sentinel hospitals in Chongqing, including weekly numbers of specimens tested positive for influenza A(H1N1)pdm09, influenza A(H3N2), influenza B, and calculated weekly positive rate using weekly number of specimens as the denominator. An influenza type or subtype was considered dominant when it accounted for at least 50% of all influenza-positive specimens.

### Estimating of influenza-associated EMRs

Given over-dispersion in mortality data, we applied negative-binomial regression models to estimate EMRs, using weekly mortality counts as the outcome and weekly proportions of specimens testing positive for influenza A(H1N1)pdm09, A(H3N2) and B as the explanatory variables.

The negative-binomial model was applied to each of the five disease categories (CRD, P&I, COPD, IHD and all-cause) and two age groups (age <65 years and ≥65 years) using a log link function. The model used was as follows:

$$\begin{array}{rl} & Y\left(t_{i}\right)=a*exp\{\beta_{0}+\beta_{1}\left(t_{i}\right)+\beta_{2}\left({t_{i}}^{2}\right)+\beta_{3}[sin(2\pi \;t_{i}/52)]\\ & \;\;\;\;\;\;\;\;\;\;\;\;\;\;\;+\beta_{4}[cos(2\pi \;t_{i}/52)]+\beta_{5}[sin(2\pi \;t_{i}/26)]\\ & \;\;\;\;\;\;\;\;\;\;\;\;\;\;\;+\beta_{6}[cos(2\pi \;t_{i}/26)]+\beta_{7}\left(A\left(H1N1\right)pdm09\right)\\ & \;\;\;\;\;\;\;\;\;\;\;\;\;\;\;+\beta_{8}\left(A\left(H3N2\right)\right)+\beta_{9}\left(B\right)+error\left(t_{i}\right)\} \end{array}$$

where Y(t_i_) is the number of death for week t_i_, *β*_0_ represents the intercept, *β*_1_ and *β_2_* represents the linear and nonlinear time trends, respectively, *β*_3_, *β*_4_, *β*_5_ and *β*_6_ account for seasonality, and *β*_7_ through *β*_9_ accounts for the percentage of specimens testing positive for week t_i_. Based on correlation between mortality outcomes and viral surveillance data at different lags (range 0–3 weeks), we used 3-week lag which had the most significant association with mortality.

The number of deaths attributable to influenza was calculated as the difference between the prediction from the full model and the predictions from the models when parameters for every influenza type/subtype were set to zero, assuming there was no influenza virus circulating. Influenza-associated excess deaths were estimated for influenza A(H1N1) pdm09, A(H3N2) and B virus, separately. EMRs associated with influenza type/subtypes were calculated by dividing the excess deaths to the registered population size. The 95% confidence intervals (*CIs*) were obtained by bootstrapping the residual 1000 times and fitting the negative-binomial regression models.^9^ The influenza-associated excess mortality rates for influenza A(H1N1) pdm09, A(H3N2) and B virus were compared based on Poisson distribution model.

All analyses were conducted using R version 3.5.1 (R Foundation for Statistical Computing, Vienna, Austria). A *P*-value of <0.05 was considered indicative of a statistically significant difference.

### Ethics approval

Study approval was obtained from the Chongqing CDC Ethics Committee.

## Results

### Annual deaths by underlying diagnosis

In the study period 2012–2018, an annual mean of 193 757 deaths were recorded in Chongqing, including 117 503 coded as CRD, 3163 coded as P&I, 33 326 coded as COPD and 23 903 coded as IHD, representing 60.6%, 1.6%, 17.2% and 12.3% of all deaths, respectively (Table 1). Adults aged ≥65 years accounted for 73.9% of all-cause deaths. The proportion of deaths coded as CRD, P&I, COPD, and IHD as well as all causes were 83.9%, 82.0%, 89.4%, 81.9%, and 85.3% for people age ≥65 years, respectively.

**Table 1.** Annual number of deaths in Chongqing, China, 2012–2018.

	All-cause		CRD^b^		P&I^c^		COPD^d^		IHD^e^	
Age group	No.^a^	Rate	No.^a^	Rate	No.^a^	Rate	No.^a^	Rate	No.^a^	Rate
**All age**										
2012	176537	605	109480	375	2874	10	32032	110	17633	60
2013	183419	623	110296	375	2881	10	31736	108	19233	65
2014	195482	658	118670	400	3179	11	34470	116	23848	80
2015	198729	664	120015	401	3106	10	34761	116	24409	82
2016	205696	682	124078	411	3515	12	34500	114	25543	85
2017	186662	612	112194	368	3117	10	31670	104	25501	84
2018	209777	682	127789	416	3467	11	34111	111	31151	101
**Total**	**1356302**	**4527**	**822522**	**2745**	**22139**	**74**	**233280**	**779**	**167318**	**557**
**Age** ≥**65**										
2012	126174	2450	89449	1737	2210	43	28203	1107	14653	285
2013	130521	3725	90566	2584	2327	66	27872	1676	16019	457
2014	142613	3937	98919	2731	2561	71	30683	1783	20404	563
2015	146034	4004	100592	2758	2541	70	31127	1804	20771	569
2016	153177	3705	104918	2538	2969	72	31025	1674	21866	529
2017	141506	3814	95758	2581	2613	70	28617	1702	22070	595
2018	162912	4007	109791	2701	2934	72	31035	1828	27012	664
Total	1002937	25642	689993	17630	18155	464	208562	11573	142795	3663
**Age**<**65**										
2012	50363	209	20031	83	664	3	3829	16	2980	12
2013	52898	204	19730	76	554	2	3864	15	3214	12
2014	52869	203	19751	76	618	2	3787	15	3444	13
2015	52695	201	19423	74	565	2	3634	14	3638	14
2016	52519	202	19160	74	546	2	3475	13	3677	14
2017	45156	169	16436	61	504	2	3053	11	3431	13
2018	46865	176	17998	67	533	2	3076	12	4139	16
**Total**	**353365**	**1363**	**132529**	**511**	**3984**	**15**	**24718**	**95**	**24523**	**94**

^a^Denotes number.

^b^Denotes circulatory and respiratory disease.

^c^Denotes pneumonia and influenza.

^d^Denotes chronic obstructive pulmonary disease.

^e^Denotes ischemic heart disease.

### Annual influenza virological surveillance

Table 2 showed the influenza surveillance data in Chongqing during 2012–2018. Overall, 27 036 specimens from ILI cases were collected for virus detection. Among tested specimens, 10.9% (2947/27036) were positive for influenza virus by reverse transcription-polymerase chain reaction. Influenza A (H1N1)pdm09, A (H3N2), and B virus comprised 23.1%, 36.6% and 40.3% of the positive influenza isolated, respectively.

**Table 2.** Annual sum of total specimens tested and specimens positive for influenza by type/subtypes in Chongqing, 2012–2018.

Year	Specimens tested	Number (%) of specimens positive for influenza	Number (%)^a^ by type/subtypes		
			A(H1N1)pdm09	A(H3N2)	B
2012	1067	483 (45.3)	1 (0.2)	299 (61.9)	183 (37.9)
2013	3855	369 (9.6)	217 (58.8)	39 (10.6)	113 (30.6)
2014	3486	458 (13.1)	42 (9.2)	273 (59.6)	143 (31.2)
2015	3700	359 (9.7)	8 (2.2)	161 (44.9)	190 (52.9)
2016	4913	515 (10.5)	26 (5.1	208 (40.4)	281 (54.6)
2017	5165	496 (9.6)	192 (38.7)	77 (15.5)	227 (45.8)
2018	4850	267 (5.5)	195 (73.0)	22 (8.2)	50 (19.7)
Total	27036	2947 (10.9)	681 (23.1)	1079 (36.6)	1187 (40.3)

^a^Proportions of subtype in total positive specimens for total positive specimens.

Influenza A(H3N2) was the predominant virus in 2012 and 2014, while A(H1N1)pdm09 predominated in 2013 and 2018, influenza B predominant in 2015 and 2016, and influenza A(H1N1)pdm09, A(H3N2) and B co-circulated in 2017 (Table 2).

**Figure 2.**
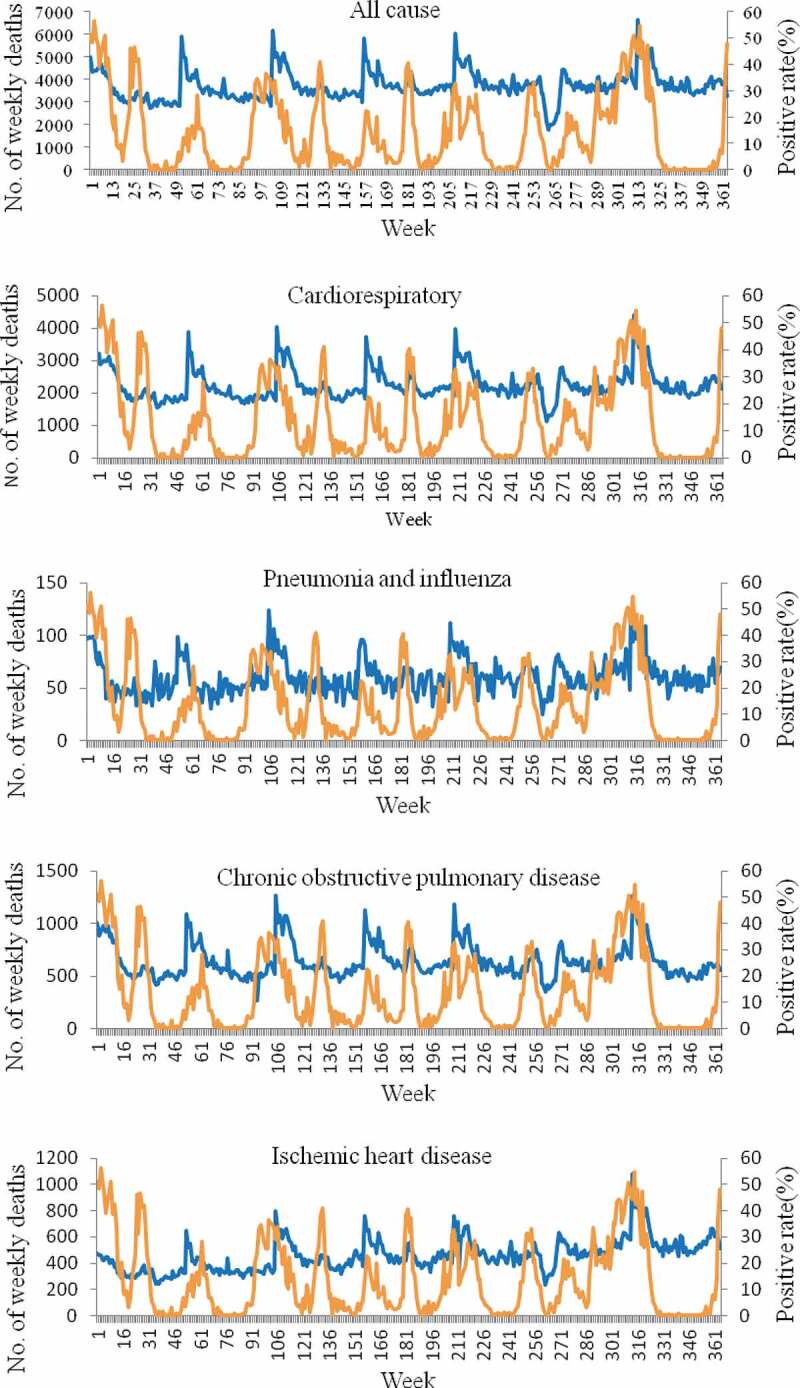
Weekly number of influenza-associated death per five causes (blue) and the proportion of influenza-positive rate (orange) in Chongqing, 2012–2018.

Weekly number of influenza-associated death per five causes and the proportion of influenza-positive in all age groups (Figure 2) showed that each of the five health outcomes has a similar pattern to that seen with influenza activity.

### Influenza-associated EMRs

Influenza was significantly associated with all health outcomes for all age groups. For people aged ≥65 years, the annual average influenza-associated EMR was 152.3(95% *CI*: 136.1–168.4) per 100 000 persons, which was significantly higher than the rate among those aged <65 years (6.8, 95% *CI*: 6.3–7.2). Age-stratified analysis revealed that influenza-associated EMRs for CRD, P&I, COPD, and IDH were more than 20-fold higher among people aged ≥65 years than among those aged <65 years (Table 3).

**Table 3.** Influenza-associated EMRs in 2012–2018 estimated by negative-binomial model.

Causes of death	No. of excess deaths per 100 000 population per year (95%*CI*)				
	A(H1N1)pdm09	A(H3N2)	B	*P*-value	Total
All cause					
< 65 y	1.0 (0.8–1.1)	0.5 (0.3–0.7)	5.3 (5.2–5.4)	0.001	6.8 (6.3–7.2)
**≥**65 y	56.5 (50.8–62.1)	13.8 (7.2–20.3)	82.03 (78.6–85.5)	<0.001	152.3 (136.1–168.4)
All ages	7.5 (6.7–8.3)	5.3 (4.5–6.1)	20.6 (20.3–21.0)	<0.001	33.5 (31.5–35.5)
CRD					
< 65 y	0.6 (0.5–0.7)	0.5 (0.4–0.6)	2.2 (2.2–2.2)	0.111	3.3 (3.1–3.6)
**≥**65 y	42.3 (38.2–46.4)	17.8 (13.0–22.6)	61.8 (59.3–64.3)	<0.001	121.9 (110.2–133.7)
All ages	5.5 (5.0–6.1)	4.97 (4.4–5.5)	14.2 (13.9–14.4)	0.001	24.7 (23.3–26.0)
P&I					
< 65 y	0.03 (0.02–0.03)	0.02 (0.01–0.03)	0.1 (0.1–0.1)	1.000	0.2 (0.1–0.2)
≥65 y	1.2 (1.1–1.4)	0. 6 (0.4–0.7)	2.1 (2.0–2.2)	0.226	3.9 (3.5–4.3)
All ages	0.2 (0.2–0.2)	0.1 (0.1–0.2)	0.5 (0.5–0.5)	1.000	0.8 (0.7–0.8)
COPD					
< 65 y	0.1 (0.1–0.2)	0.2 (0.2–0.2)	0.5 (0.5–0.5)	0.144	0.8 (0.8–0.9)
≥65 y	14.0 (12.6–15.3)	7.9 (6.3–9.5)	23.1 (22.3–24.0)	0.001	45.0 (41.0–48.9)
All ages	1.8 (1.6–1.9)	1.9 (1.7–2.0)	4.9 (4.8–5.0)	0.037	8.5 (8.1–9.0)
IHD					
< 65 y	0.2 (0.2–0.2)	0 (−0.02–0.02)	0.3 (0.3–0.3)	1.000	0.5 (0.4–0.5)
≥65 y	12.0 (11.1–12.8)	5.5 (4.5–6.5)	11.1 (10.6–11.6)	0.396	28.6 (26.1–31.0)
All ages	1.7 (1.6–1.8)	1.1 (1.0–1.2)	2.3 (2.2–2.3)	0.401	5.0 (4.7–5.3)

Compared with influenza A virus, influenza B virus showed a significantly higher mortality burden. Table 3 showed that for all age group influenza B virus accounted for the highest EMR of all-cause mortality (20.6, 95% *CI*: 20.3–21.0), followed by A(H1N1)pdm09 (7.5, 95% *CI*: 6.7–8.3) and A(H3N2) (5.3, 95% *CI*: 4.5–6.1) per 100 000 persons. A similar pattern was also observed for CRD, P&I, COPD and IDH among both those aged ≥65 years and those aged <65 years.

## Discussion

This study estimated the mortality burden of seasonal influenza in Chongqing based on robust vital statistics and mortality data during 2012–2018. We estimated an average of 10 025 influenza-associated all causes deaths per year, accounting for 5.2% of all reported deaths from the death registration system. All-cause mortality rate associated with influenza was 33.5 (95% *CI*: 31.5–35.5) per 100 000 persons, and in separate cause-specific models we attributed 24.7 (95% *CI*: 23.3–26.0), 0.8 (95% *CI*: 0.7–0.8), 8.5 (95% *CI*: 8.1–9.0), and 5.0 (95% *CI*: 4.7–5.3) per 100 000 persons EMRs to CRD, P&I, COPD and IDH, respectively.

Our estimates were much higher than estimates from Beijing,^10^ Hefei,^11^ Yancheng,^12^ Guangzhou^13^ in China and other countries such as Thailand,^14^ United States,^15^ Singapore, and New Zealand.^16,17^ For example, a study carried out in Beijing reported that an average of 17.2 (95% *CI*, 7.2–67.5) per 100 000 persons for all-cause death, 13.5 (95% *CI*, 5.8–51.7) per 100 000 persons for CRD excess mortality associated with influenza annually from 2007 to 2013.^10^ Another study conducted by China CDC reported that an average of 11.3 (95% *CI*, 1.4–50.4) per 100 000 persons for all-cause death, 7.8 (95% *CI*, 1.8–50.4) per 100 000 persons for CRD, 0.5 (95% *CI*, 0.1–2.3) per 100 000 persons for P&I, 1.0 (95% *CI*, 0.1–7.0) per 100 000 persons for IDH and 3.0 (95% *CI*, 0.7–12.0) per 100 000 persons for COPD excess mortality associated with influenza annually in five south cities (Shanghai, Wuhan, Yichang, Ningbo, and Guangzhou) from 2003 to 2008.^5^ The possible explanations for the differences might be associated with the regional variation in socioeconomic and demographic factors, different models used and different study periods. Furthermore, differences in successful implementation of seasonal influenza vaccination might also have played an important role. The influenza vaccination subsidy policy was quite different in different regions. Many high-income countries conducted influenza vaccination program in older adults with the goal of reducing the influenza-associated mortality in this high-risk group. China in general has low vaccination coverage in the population,^18^ and only in a few regions such as Beijing where the local government fully subsidizes the vaccination in older adults.^19^ At present, the cost of influenza vaccination is borne using the surplus fund of basic social medical insurance for urban residents individual accounts in Chongqing and many people have to pay for the seasonal influenza vaccine out of pocket,^18^ which might lead to very low influenza vaccination coverage in this area.

Overall, the impact of influenza on mortality in Chongqing disproportional affected people aged ≥65 years, which is consistent with the findings of previous studies.^5,16,20–22^ According to the 2010 census results in China, the proportion of people ≥65 years old in Chongqing was the highest in China, which has reached the average level of developed countries.^23^ Given the high excess mortality among elderly people and the serious aging problem in Chongqing, great efforts should be made to increase seasonal influenza vaccination coverage in this population.

It is not surprising that CRD was the major contributor to total influenza-associated deaths, as CRD was the primary cause in Chongqing. Our data also suggested a significant association between influenza and COPD death. This finding was not unexpected given multiple reports relating the association between COPD death and seasonal influenza. The relation may be explained by the dysfunction of innate immune defenses and destruction of the lung parenchyma or airway remodeling by acute virus infections.^24^ In China, COPD was the third leading cause of death and accounted for more 965.9 thousand deaths in 2017.^25^ There was accumulating evidence suggesting that influenza vaccination was associated with a reduced risk of mortality in COPD patients and seasonal influenza vaccination was recommended by international and national health organizations; nonetheless coverage remains sub-optimal compared to recommended targets.^26^

Notably, our study observed that influenza B caused the highest-burden compared with A(H1N1)pdm09 and A(H3N2). This mortality pattern is consistent with those described in studies conducted by China CDC,^5^ Beijing CDC and Guangzhou CDC,^6,10^ but differs from studies conducted in other regions and countries such as Yancheng,^12^ Hefei,^11^ Hong Kong,^9^ Singapore,^16^ South Korea,^27^ United States,^15^ and New Zealand,^17^ where the highest EMR was associated with influenza A(H3N2). The inconsistent severity profile of influenza type/subtypes warrants further investigation in more locations in future studies.

Despite providing insightful estimates of influenza-associated disease burden in Chongqing, this study has several limitations. Firstly, this was an ecological study with aggregated data, so ecologic fallacy was inevitable. Secondly, it was not possible to adjust for other co-circulating respiratory viruses such as respiratory syncytial virus, adenovirus, and parainfluenza virus, which may have confounded the results. In future, the establishment of such surveillance system may improve the accuracy of influenza-associated mortality burden. Thirdly, our results were dependent on the coding and registration of deaths, errors including possible underreporting and misclassification of deaths could lead to the underestimation of influenza-associated excess mortality, especially for disease-specific mortality indicators. Finally, we only examined the impact of influenza epidemics on excess mortality. In future, a more comprehensive assessment including hospitalization, year-of-life lost and economic burden of influenza would help to fully assess the disease burden of influenza in Chongqing.

## Conclusions

Our study demonstrated a substantial influenza-related mortality burden in the largest municipality in China from 2012 to 2018, primarily associated with CRD and COPD deaths in the elderly. Influenza B virus caused a relatively higher excess mortality impact compared with A (H1N1)pdm09 and A(H3N2). This study supported the recent recommendation by the National Immunization Advisory Committee on “Technical guidelines for seasonal influenza vaccination in China (2018–2019)” for elderly people and individuals with chronic underlying conditions, such as COPD, CVD or diabetes, be treated as priority groups for seasonal influenza vaccination.^28^ It is advisable to optimize future seasonal influenza vaccine reimbursement policy in Chongqing to curb disease burden.
